# Supplementary material for: The interactome of intact mitochondria by cross-linking mass spectrometry provides evidence for coexisting respiratory supercomplexes
Source: Mol Cell Proteomics. 2017 Dec 8;17(2):216–32. doi: 10.1074/mcp.RA117.000470 (PMC5795388; doi:10.1074/mcp.RA117.000470)
Supplement: Supplemental Data [file supp_RA117.000470_133922_0_supp_23978_fzffwf.zip › spectra_annotation/mito_spectra_annotation/247-1-11-1-3-1.pdf]

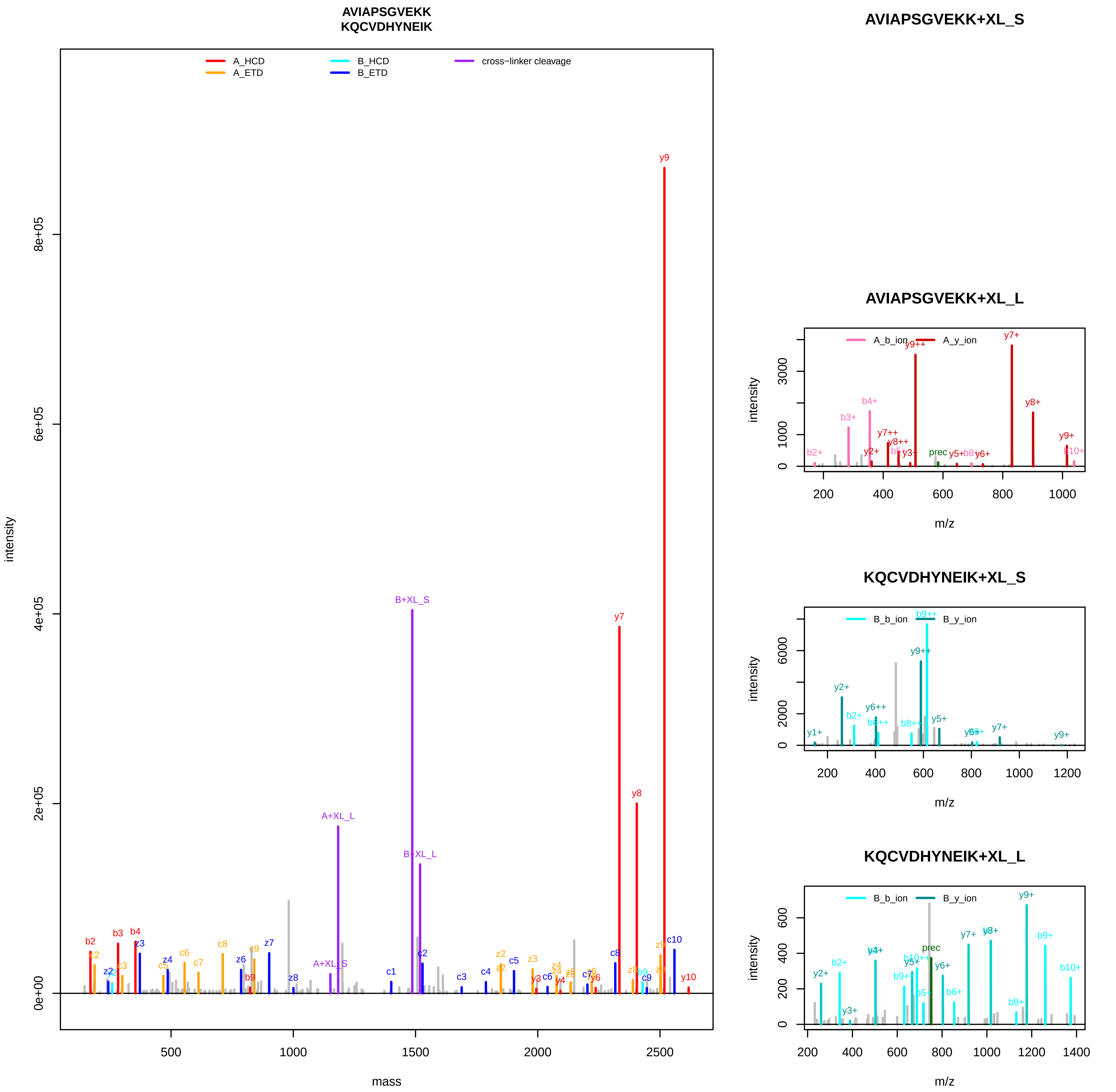
AVIAPSGVEKK
KQCVDHYNEIK
A_HCD
A_ETD
B_HCD
B_ETD
cross−linker cleavage
intensity
mass
0e+00
2e+05
4e+05
6e+05
8e+05
500
1000
1500
2000
2500
y9
y7
y8
B+XL_S
B+XL_L
A+XL_L
A+XL_S
y10
c10
b2
b3
b4
c2
z2
c3
z3
z4
c5
c6
c7
c8
z6
c9
z7
z8
b9
c1
c3
c4
c5
c6
c7
c8
c9
z5
y3
y4
y6
AVIAPSGVEKK+XL_S
AVIAPSGVEKK+XL_L
A_b_ion
A_y_ion
intensity
m/z
y7+
y9++
y8+
b4+
b3+
y7++
y8++
y9+
b10+
b2+
y2+
y3+
prec
y5+
b8+
y6+
KQCVDHYNEIK+XL_S
B_b_ion
B_y_ion
b9++
y9++
y2+
y6++
b2+
y5+
y1+
y7+
y9+
KQCVDHYNEIK+XL_L
y9+
y8+
y7+
b9+
b10+
y4+
b2+
y2+
prec
y6+
y5+
b9++
b6+
b8+
y3+
200
400
600
800
1000
1200
1400
0
1000
3000
2000
6000
